# An Integrated Service Delivery Model to Identify Persons Living with HIV and to Provide Linkage to HIV Treatment and Care in Prioritized Neighborhoods: A Geotargeted, Program Outcome Study

**DOI:** 10.2196/publichealth.4675

**Published:** 2015-10-08

**Authors:** Paula M Frew, Matthew Archibald, Jay Schamel, Diane Saint-Victor, Elizabeth Fox, Neena Smith-Bankhead, Dazon Dixon Diallo, Marcia M Holstad, Carlos del Rio

**Affiliations:** ^1^ Division of Infectious Diseases Department of Medicine Emory University School of Medicine Atlanta, GA United States; ^2^ Hubert Department of Global Health Rollins School of Public Health Emory University Atlanta, GA United States; ^3^ Department of Sociology Colby College Waterville, ME United States; ^4^ AID Atlanta Atlanta, GA United States; ^5^ SisterLove, Inc. Atlanta, GA United States; ^6^ Emory University School of Nursing Atlanta, GA United States

**Keywords:** human immunodeficiency virus, human immunodeficiency virus prevention, human immunodeficiency virus testing, racial/ethnic minorities, community-based organizations, High-Impact Prevention

## Abstract

**Background:**

Recent studies have demonstrated that high human immunodeficiency virus (HIV) prevalence (2.1%) rates exist in “high-risk areas” of US cities that are comparable to rates in developing nations. Community-based interventions (CBIs) have demonstrated potential for improving HIV testing in these areas, thereby facilitating early entry and engagement in the HIV continuum of care. By encouraging neighborhood-based community participation through an organized community coalition, Project LINK sought to demonstrate the potential of the CBI concept to improve widespread HIV testing and referral in an area characterized by high poverty and HIV prevalence with few existing HIV-related services.

**Objective:**

This study examines the influence of Project LINK to improve linkage-to-care and HIV engagement among residents of its target neighborhoods.

**Methods:**

Using a venue-based sampling strategy, survey participants were selected from among all adult participants aged 18 years or more at Project LINK community events (n=547). We explored multilevel factors influencing continuum-of-care outcomes (linkage to HIV testing and CBI network referral) through combined geospatial-survey analyses utilizing hierarchical linear model methodologies and random-intercept models that adjusted for baseline effect differences among zip codes. The study specifically examined participant CBI utilization and engagement in relation to individual and psychosocial factors, as well as neighborhood characteristics including the availability of HIV testing services, and the extent of local prevention, education, and clinical support services.

**Results:**

Study participants indicated strong mean intention to test for HIV using CBI agencies (mean 8.66 on 10-point scale [SD 2.51]) and to facilitate referrals to the program (mean 8.81 on 10-point scale [SD 1.86]). Individual-level effects were consistent across simple multiple regression and random-effects models, as well as multilevel models. Participants with lower income expressed greater intentions to obtain HIV tests through LINK (*P*<.01 across models). HIV testing and CBI referral intention were associated with neighborhood-level factors, including reduced availability of support services (testing *P*<.001), greater proportion of black/African Americans (testing and referral *P*<.001), and reduced socioeconomic capital (testing *P*=.017 and referral *P*<.001). Across models, participants expressing positive attitudes toward the CBI exhibited greater likelihood of engaging in routine HIV testing (*P*<.01) and referring others to HIV care (*P*<.01). Transgender individuals indicated greater intent to refer others to the CBI (*P*<.05). These outcomes were broadly influenced by distal community-level factors including availability of neighborhood HIV support organizations, population composition socioeconomic status, and high HIV prevalence.

**Conclusions:**

Project LINK demonstrated its potential as a geotargeted CBI by evidencing greater individual intention to engage in HIV testing, care, and personal referrals to its coalition partner organizations. This study highlights important socioecological effects of US-based CBIs to improve HIV testing and initiate acceptable mechanisms for prompt referral to care among a vulnerable population.

## Introduction

### Background

Community-based interventions (CBIs) are a feasible, sustainable approach to increase widespread human immunodeficiency virus (HIV) testing and improve entry and engagement in the HIV continuum of care [1,2]. Ideally, engagement in care is a seamless, coordinated process commencing with individual testing, diagnosis, and treatment initiation. Yet those at highest risk of HIV infection are the most challenging to engage and susceptible to delays across the care continuum. HIV testing delay is frequent in US men-who-have-sex-with-men (MSM) populations, with an estimated 19-26% of MSM unaware of their status [3]. Delayed testing is associated with a lack of awareness or denial of perceived risk for infection, age, and race/ethnicity [3,4]. Racial and ethnic minorities are at increased risk of delayed referral to HIV care and treatment following diagnosis [5,6]. Rates of delayed testing rates remain high; in 2013, 23.6% of newly diagnosed HIV patients in the United States were classified as Stage 3 (acquired immune deficiency syndrome, AIDS) at diagnosis [7]. Treatment delay is more common among black/African Americans, immigrants, and uninsured individuals [8]. Between 20% and 40% do not link to HIV care within a year of diagnosis, a delay which is associated with higher rates of virologic failure, increased morbidity and mortality, and immune system damage resulting from delayed receipt of antiretrovirals [9].

Recent studies have demonstrated that high HIV prevalence (2.1%), comparable with HIV rates in developing nations, are present in “high-risk areas” of US cities, particularly in neighborhoods characterized by high poverty and HIV prevalence [10]. These “hot spot” areas are experiencing local, yet generalized, HIV microepidemics. Notably, many of these areas are located within 12 major metropolitan areas that account for approximately 44% of all estimated AIDS cases, signifying the challenges facing continuum of care access and delivery [11]. Thus, it is important to look at health care service delivery in these areas and examine the extent to which these options are culturally compatible and socially sensitive to the needs of those who could most benefit from geographically targeted HIV prevention and care. Previous studies have identified the importance of recognizing the spatial distribution of HIV burden [11,12], HIV service provision and continuum of care objectives [13-15], and also of the spatial and ecosocial dimensions of the development and delivery of CBIs targeting HIV transmission [1,16,17].

### Project LINK

Project LINK was an initiative supported by the Atlanta AIDS Partnership Fund and the Community Foundation of Greater Atlanta to increase HIV testing in an area of Atlanta, Georgia, characterized by high poverty and HIV prevalence. LINK’s goals were to identify residents living with HIV and directly connect those living with HIV to appropriate medical care and treatment programs. In addition, LINK created a model for building lasting partnerships between community and HIV/AIDS outreach agencies. The project was initiated as a result of numerous meetings with community partners and local residents concerned about the high HIV prevalence rate in their neighborhood. Collectively, all parties reviewed HIV/AIDS data, held discussions on community needs and assets, and worked to identify specific strengths and potential contributions of the selected agency partners to the delivery of HIV prevention and care in the selected neighborhoods.

Development of the intervention thus occurred through a process of community-based participatory research [18]. Community members and leaders were invited to attend a series of meetings with the funder, technical advisors, and evaluative team to discuss factors that may be influencing high HIV prevalence rates in the target neighborhoods for the intervention, a process that has proven to be effective in eliciting critical intervention points [19]. These conversations led to an inventory of structural, social, and individual-level factors that aligned well with the socioecological model [20]. Thus, the intervention was informed by this theoretical framework based on community consensus and resulting activities that focused on addressing such factors across levels. As a result of these planning activities, 5 community partner agencies were selected to collaborate with the selected community to increase the capacity of local residents and agencies to conduct HIV prevention and linkage-to-care activities: the Center for Black Women’s Wellness, Inc (CBWW), the Atlanta Harm Reduction Coalition (AHRC), AID Atlanta, Positive Impact, and SisterLove, Inc.

High-Impact Prevention [21], an intervention approach adopted by the Centers for Disease Control and Prevention (CDC) in 2013 to improve the effectiveness of HIV prevention efforts, maximizes the relevance of proposed interventions through consideration of the cost effectiveness and feasibility of full-scale implementation, as well as assessment of coverage, interactions, and combinations. Project LINK utilized this same strategy in bringing together various types of service providers while supporting partnership development and linkage systems to ensure that those in need of HIV services had access to a comprehensive range of resources appropriate for their needs, accessible to those at greatest risk. Each of the participating agencies had a long history of providing culturally competent programs and integrative service delivery for HIV/AIDS prevention, testing, counseling, and linkage to HIV case managers and mental health services. Each agency had a specific role related to HIV testing and subsequent coordinated referral and linkage to in-house clinical care. In addition, supportive programs such as the evidence-based “HealthyLove” HIV prevention party (SisterLove) [22,23]; needle exchange (AHRC) [24,25]; women’s support groups (CBWW, SisterLove, and AID Atlanta) [26]; and mental health counseling and domestic violence prevention and response training (Positive Impact) [27] were components of the agencies’ service delivery package. These were offered to residents at neighborhood schools, churches, housing complexes, and community meeting locations, in addition to street outreach conducted by the agencies’ mobile units.

The community partners developed Project LINK to normalize routine HIV testing and to provide coordinated linkage to HIV care among the participating partner entities serving residents in the target neighborhoods. LINK focused on engaging residents living in an area comprising 2 target zip codes within a generalized HIV high-prevalence cluster (≥1.0% HIV case prevalence) and few established HIV continuum-of-care resources [12]. For this study, we also identified a secondary catchment area of 5 adjacent zip codes within the cluster, directly abutting the 2 target zip codes. The initiative sought to reach about 5000 residents over a 1-year period.

## Methods

### Study Design and Sample

The purpose of this study is to assess the influence of the Project LINK CBI to improve linkage-to-care and HIV engagement in a selected area of Atlanta that demonstrated a high HIV prevalence burden and limited health care services in its census tracts. Individuals enrolled in this study were selected from survey sessions that were randomly scheduled over a 10-month demonstration period. This resulted in questionnaire collection at 31 unique LINK-organized community activities and programs during this period. Outcomes related to intention to utilize CBI HIV testing resources and to refer other CBIs were included in surveys administered at 23 of these postimplementation activities within the process evaluation phase of the CBI. Eligibility criteria specified inclusion of men and women aged 18 and over who had the ability to read and write English. Respondents selected a gift card, t-shirt, transit card, or some other health-promotion item for their participation. The Emory University Institutional Review Board approved the protocol (00005278) prior to study inception.

### Study Measures

The questionnaire included items on HIV testing and referral to the CBI, in addition to sociodemographics, perceptions, and attitudinal factors. Our primary outcome of interest was participants’ willingness to engage in routine HIV testing through the CBI, measured by the item, "On a scale from 0 (definitely not) to 10 (definitely so), rank your likelihood of getting your next HIV test with a LINK agency in the next 6 months." To assess the potential of the CBI to motivate participants to refer others to its services, we included the item, "On a scale from 0 (definitely not) to 10 (definitely so), rank your likelihood of getting others involved in Project LINK."

### Individual-Level Factors

We estimated the impact of multiple, nested influential factors on the CBI’s key outcomes of interest, HIV testing, and LINK service referral. Demographic factors from survey responses included race, income, gender, and age. For the purposes of this study, race was coded as "white" and "nonwhite." Household income responses included 6 categories in US $20,000 increments from “less than US $20K per year” to “over US $100K per year,” and were treated as a continuous variable for our analyses. Gender was included in our models as a three-level categorical variable with levels "male," "female," and "transgender." Age was measured in years, and was incorporated into our models as a continuous covariate.

### Exploratory Factor Analysis

Psychometric items were drawn from previous HIV behavioral research demonstrating excellent internal consistency and validity of items [20,28]. The questionnaire included measures assessing attitudes about the LINK initiative, HIV/AIDS, social norms, and community involvement [1,29,30]. Subscale development utilized principal component analyses with varimax rotation, followed by assessment of components’ internal consistency. Because some item responses were missing (3.6%), we conducted Little’s Missing Completely at Random (MCAR) test to analyze the overall pattern on surveys and subsequently performed mean imputation.

### Neighborhood-Level Factors

To assess the CBI’s success in promoting linkage to HIV support in underserved communities, we explored the relationship between LINK service utilization and the availability of HIV services, support, and educational organizations. Previous research provided a comprehensive catalog of HIV service offerings in both the 2 CBI target area zip codes and the surrounding neighborhoods [12]. Using their reported residential zip code, each participant was connected to the total number of discrete, permanent HIV-related services available in their community [13]. Discrete services included HIV case management, HIV medical treatment/services, HIV testing and counseling, community education and outreach, mentoring and support services, etc. and separate services from the same provider were counted separately. Only services provided prior to the initiation of Project LINK were included in this count.

HIV prevalence was estimated for each zip code using census-tract-level HIV diagnosis counts from 2005 to 2007. These census-tract-level HIV counts were aggregated to zip-code-level counts using Esri ArcGIS version 10.2 [31]. Counts from census tracts overlapping more than 1 zip code were split by area. HIV prevalence was computed by dividing the aggregate HIV diagnosis count by the zip code population, as measured in the US Census 2000 [32].

Other neighborhood-level factors were included to reflect the socioeconomic composition of the community. These variables included the proportion of black/African American residents, the proportion of residents aged 25 years or more, the proportion of male residents over 18 who have graduated high school, median income, male employment rate, and the proportion of vacant households. These community characteristics were obtained at the zip code level from the US Census Bureau's Census 2000 [32].

### Statistical Analyses

We first computed descriptive statistics for characteristics of our sample of CBI participants and for questions eliciting participant impressions of the CBI. We then computed descriptive statistics for our 2 outcome measures, willingness to engage in routine HIV testing through the CBI, and intention to refer others to the CBI. To compare these outcomes between participants living in the 2 primary target zip codes, those living in the 5 secondary catchment zip codes, and those living outside the target areas, we utilized analysis of variance (ANOVA) post hoc pairwise analysis with Tamhane adjustment.

Next, we employed random-intercept linear mixed models to examine the effect of individual- and neighborhood-level covariates on CBI utilization and referral outcomes. Baseline differences among participants from different zip codes were adjusted for through the incorporation of random intercepts for each zip code. To focus analysis on effects relating to individuals within the CBI area, only participants from the 2 target and 5 secondary catchment zip codes were included in the multilevel analysis. Because 7 zip codes did not admit multiple neighborhood effects in a single model, separate models were fit for each neighborhood-level covariate, each regressing a single neighborhood-level covariate and all individual-level covariates on a CBI outcome. To assess the stability of individual-level effects, multiple linear and random-intercept (by zip code) models were also fit using only the individual and psychosocial variables, excluding neighborhood-level variables. Random-intercept models used the *xtreg* procedure with maximum likelihood estimation in Stata version 13 [33]. Participants with missing outcome responses were excluded by listwise deletion. Variance inflation factors were used to assess all models for multicollinearity; no issues were discovered. For all hypothesis tests, results were considered statistically significant if *P*<0.05.

## Results

### Sample Characteristics

Of the 597 respondents selected at the 23 postimplementation activities, 414 (69%) lived within the 2 primary LINK target zip codes, 37 (6.2%) within the 5 secondary catchment zip codes, 101 (17%) lived outside the targeted area, and 45 (7.5%) did not list a home zip code. Table 1 describes the sociodemographic characteristics of the sampled participants, together with the characteristics of the participants living within the 2 target zip codes and the 5 secondary catchment zip codes (Table 1). The CBI participants included a majority of black/African American (88.8%, n=530) participants in the age range of 40-59 years (63.7%, n=380; Table 1). Respondents were evenly split between male and female participants (47.6%, n=284 versus 45.2%, n=270). In addition, the sample included 27 transgender persons (the majority male-to-female). Most respondents obtained high-school diplomas or general educational developments (56.8%, n=339), yet many were also unemployed (54.6%, n=326) and had annual household income less than US $20,000 per year (78.2%, n=467).

**Table 1:** Participant sociodemographic characteristics.^a^

		All respondents	Target area and secondary catchment
		Frequency (%)	Frequency (%)
**Age** ^b,c^			
	18-29 years	78 (13.1)	56 (12.4)
	30-39 years	85 (14.2)	58 (12.9)
	40-49 years	213 (35.7)	175 (38.8)
	50-59 years	167 (28.0)	133 (29.5)
	≥60 years	35 (5.9)	23 (5.1)
	*Missing*	*19 (3.2)*	*6 (1.3)*
**Gender**			
	Male	284 (47.6)	218 (48.3)
	Female	270 (45.2)	207 (45.9)
	Transgender: M to F	22 (3.7)	15 (3.3)
	Transgender: F to M	5 (0.8)	3 (0.7)
	*Missing*	*16 (2.7)*	*8 (1.8)*
**Race**			
	White	29 (4.9)	12 (2.7)
	Nonwhite	531 (88.9)	415 (92.0)
	*Missing*	*37 (6.2)*	*43 (9.5)*
**Ethnicity**			
	Asian/Asian American/Pacific Islander	11 (1.8)	5 (1.1)
	Hispanic/Latino/Chicano	1 (0.2)	0 (0.0)
	African American/black	530 (88.8)	421 (93.3)
	Caucasian/white	20 (3.4)	6 (1.3)
	American Indian/Alaska Native	6 (1.0)	3 (0.7)
	Multiracial/Multicultural	10 (1.7)	7 (1.6)
	*Missing*	*19 (3.2)*	*9 (2.0)*
**Sexual orientation**			
	Heterosexual	512 (85.8)	400 (88.7)
	LGBTQQ^d^	69 (11.6)	42 (9.3)
	*Missing*	*16 (2.7)*	*9 (2.0)*
			
**Relationship status**			
	Single	387 (64.8)	299 (66.3)
	Married/domestic partner	105 (17.6)	67 (14.9)
	Divorced/separated	69 (11.6)	60 (13.3)
	Widowed	26 (4.4)	21 (4.7)
	*Missing*	*10 (1.7)*	*4 (0.9)*
			
**Educational attainment**			
	K-8 grade	71 (11.9)	52 (11.5)
	High-school graduate/general educational development	339 (56.8)	282 (62.5)
	Technical/Vocational or Associates	100 (16.8)	81 (18.0)
	Bachelor degree	39 (6.5)	16 (3.5)
	Master's degree	14 (2.3)	3 (0.7)
	Doctorate	9 (1.5)	1 (0.2)
	*Missing*	*25 (4.2)*	*16 (3.5)*
			
**Household income**			
	Less than US $20,000	467 (78.2)	395 (87.6)
	US $20,001-US $40,000	43 (7.2)	25 (5.5)
	US $40,001-US $60,000	19 (3.2)	5 (1.1)
	US $60,001-US $80,000	16 (2.7)	4 (0.9)
	US $80,000-US $100,000	9 (1.5)	1 (0.2)
	More than US $100,000	17 (2.8)	4 (0.9)
	*Missing*	*26 (4.4)*	*17 (3.8)*
**Employment status**			
	Employed full time	91 (15.2)	39 (8.6)
	Employed part-time	95 (15.9)	80 (17.7)
	Unemployed	326 (54.6)	279 (61.9)
	Other^e^	73 (12.2)	49 (10.9)
	*Missing*	*12 (2.0)*	*4 (0.9)*
**Distance traveled to CBI activity**			
	<5 miles	459 (76.9)	388 (86.0)
	6-9 miles	57 (9.5)	33 (7.3)
	10-20 miles	37 (6.2)	10 (2.2)
	>20 miles	31 (5.2)	14 (3.1)
	*Missing*	*13 (2.2)*	*6 (1.3)*

^a^The italics are used to emphasize the percentage of nonresponders for each item

^b^Mean and SD for all respondents: 44.6 and 11.4, respectively.

^c^Mean and SD for respondents in target area and secondary catchment: 44.9 and 11.0, respectively.

^d^Lesbian, gay, bisexual, transgender, queer, and questioning

^e^Retired, student, self-employed, disability, and illicit

Individuals in our sample provided insight on their motivations to attend the LINK activities (Table 2). Most common reasons provided were to obtain medical and scientific information (36.5%, n=218) and meeting others who share similar HIV/AIDS concerns in the community (25.3%, n=151). A majority of respondents also expressed strong approval of LINK activities (64.0%, n=382) and 57.0% rated Project LINK as excellent/outstanding (n=340).

**Table 2:** Participant CBI impressions.^a^

		All respondents (n=597)	Target area and secondary catchment (n=451)
		Frequency (%)	Frequency (%)
**Motivation to attend CBI activity**			
	Get the latest scientific/medical information	218 (36.5)	168 (37.3)
	Meet others who share my concerns	151 (25.3)	119 (26.4)
	Sense of obligation to my community	88 (14.7)	58 (12.9)
	Learn about volunteer opportunities	58 (9.7)	46 (10.2)
	Other^b^	52 (8.7)	42 (9.3)
	*Missing*	*30 (5.0)*	*18 (4.0)*
**Rating of this event/activity**			
	Excellent/outstanding	382 (64.0)	302 (67.0)
	Good/very good	179 (30.0)	131 (29.0)
	Fair/poor	8 (1.3)	6 (1.3)
	No opinion	10 (1.7)	4 (0.9)
	*Missing*	*18 (3.0)*	*8 (1.8)*
**Overall impression of Project LINK**			
	Excellent/outstanding	340 (57.0)	268 (59.4)
	Good/very good	226 (37.9)	167 (37.0)
	Fair/poor	6 (1.0)	5 (1.1)
	No opinion	15 (2.5)	8 (1.8)
	*Missing*	*10 (1.7)*	*3 (0.7)*

^a^The italics are used to emphasize the percentage of nonresponders for each item.

^b^Multiple responses given included write-in comments such as accompanying friend/relative, treatment/testing, compensation, life change, and community service.

The 2 primary CBI target zip codes and 5 secondary catchment zip codes differed in the availability of HIV continuum-of-care services. Within 1 primary target zip code, there were no HIV services available to residents. The other primary zip code had 10 HIV services located within the area (eg, testing, support). The number of available HIV services identified within the 5 secondary target zip codes ranged from only 3 or 4 in 3 of the zip code areas to 49 in 1 zip code area.

### Psychosocial Factors

Psychosocial subscale items and results of the exploratory factor analysis are detailed in Table 3. Chosen subscales include *LINK Attitudes* about the risks of HIV and benefits of LINK involvement, degree of psychological *LINK Engagement*, *Negative Participatory Norms* associated with HIV testing and the CBI, *Perceived LINK Social Support*, and *Identification with LINK Brand* (Table 3). The scales exhibited excellent psychometric properties including strong internal consistencies across domains (alpha=.733-.940). Responses to the items were rated on a 5-point scale and subscale scores were summed; higher scores indicated higher levels of the attribute.

**Table 3:** Factor subscales for psychosocial domains.

Factor	Factor characteristics and questions	Loading
LINK attitudes^a^		
	I benefit from Project LINK services.	.77
	I like getting involved with Project LINK.	.76
	My community will really benefit from Project LINK.	.73
	My involvement will improve my community's trust in Project LINK.	.73
	My involvement in Project LINK will improve my health.	.73
	My participation in Project LINK would be very good.	.72
	I would participate in Project LINK activities because it would help to prevent AIDS.	.72
	I feel that my involvement in Project LINK is making an important difference.	.70
	HIV testing is a benefit of getting involved.	.68
	HIV is a serious concern in my immediate community.	.68
LINK engagement^b^		
	Getting involved in the Project LINK effort is liberating.	.77
	Project LINK is a social justice effort.	.74
	Project LINK will reduce health disparities.	.70
	I feel a sense of purpose in this cause.	.69
	It is fun to be involved with the Project LINK.	.68
	I feel a sense of belonging through my participation in this effort.	.67
	My involvement is helping to protect the rights of others.	.67
	I am advancing the public's health and well-being through my support of this cause.	.65
	I am as source of HIV/AIDS information in my community.	.62
	Being involved with the Project LINK helps me to feel empowered.	.59
	I experience a sense of community in this cause.	.59
	I would be very concerned about the outcome of any effort of which I am affiliated.	.42
	It is extremely important to make the right choice in selecting a volunteer organization.	.40
	The Project LINK effort is very different from others.	.40
Negative participatory norms^c^		
	I think my friends would negatively judge me if I sought HIV testing.	.84
	I tend to be worried about what people think of me, even if I do not know them.	.79
	Participating in Project LINK seems risky.	.75
	I think some of my family members would be upset if I participated in Project LINK.	.72
	People negatively judge those who participate in Project LINK.	.70
	Even if I wanted to participate in Project LINK, I just do not have the time.	.69
	I generally do what my family expects of me.	.58
	If people heard of my participation with the Project LINK, they would form an opinion of me.	.57
	In general, I am among the last of my circle of friends to try new things.	.55
LINK social support^d^		
	If I decided to participate in Project LINK, I probably would tell my partner.	.61
	I would do something even if members of my social group disagreed with my actions.	.56
	I think my work colleagues would approve of my involvement.	.54
	Most people important to me think my involvement in Project LINK is good.	.50
	I think my doctor would approve of my involvement in Project LINK.	.49
	My immediate family is supportive of my involvement in Project LINK.	.48
	If my pastor supported Project LINK, I would be inclined to get involved.	.42
LINK brand perception^e^		
	Prior to joining any organization, I prefer to consult a friend who has experience with that group.	.64
	Hearing that somebody else is involved with the Project LINK tells me a lot about that person.	.58
	When it comes to deciding whether to join a new organization, I rely on experienced friends or family members for advice.	.56
	Being active with the Project LINK would help me to express who I am.	.47
	You can tell a lot about a person by their community affiliations.	.37

^a^Alpha=0.940; 10 items

^b^Alpha=0.935; 14 items

^c^Alpha=0.880; 9 items

^d^Alpha=0.830; 7 items

^e^Alpha=0.733; 5 items

### Linkage to HIV Testing

Most participants felt comfortable obtaining an HIV test with designated LINK providers, as indicated by high mean intention to test for HIV using a LINK agency (mean 8.66 on 10-point scale [SD 2.51]). One-way ANOVA by residence within CBI target areas (primary, secondary, outside of target area) found statistically significant differences among persons living in the LINK target area, those living adjacent to the primary intervention zone, and those coming from outside the designated zip codes with desire to use CBI HIV testing resources (*F*_2,447_=11.6, *P*<.001). Tamhane post hoc analyses indicated that respondents living in the 2 CBI target zip codes expressed greater intention to engage in routine HIV testing through the CBI compared with those living outside the target and secondary catchment zip codes (difference=1.6, *P*=.004).

**Figure 1:**
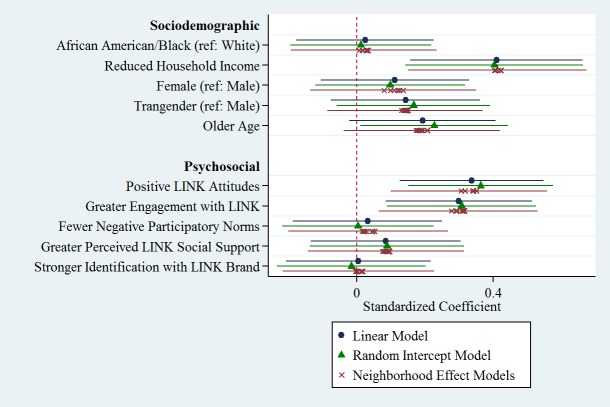
Individual predictors of HIV Testing.

**Figure 2:**
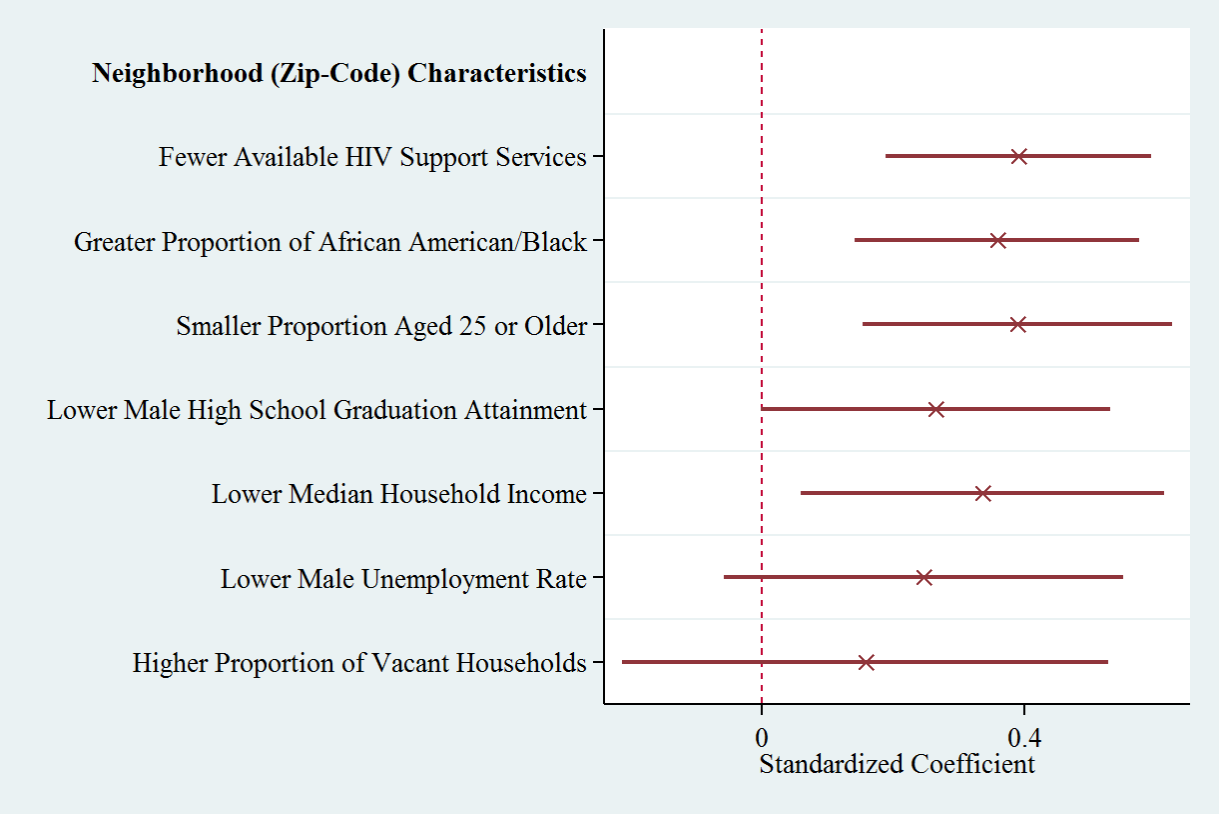
Adjusted neighborhoods predictors of HIV Testing.

The results of the multiple regression and random-intercept models with individual-level covariates are presented in Table 4 and model parameters in Table 5. Figure 1 shows the individual predictors of HIV testing. The multiple regression model incorporates all individual and psychosocial independent variables (race, income, gender, age, and the 5 psychosocial scales), but no neighborhood-level factors. The individual-level random-intercept model adds a random intercept for zip code to the previous model. Results from the multilevel models containing all individual-level covariates and a single neighborhood-level covariate are detailed in Table 6. Figure 2 shows the adjusted neighborhoods predictors of HIV testing. Full results for individual and psychosocial effects in the multilevel models are given in Multimedia Appendix 1. The coefficient estimates for individual and psychosocial covariates are very similar across multivariable models (Tables 4 and 6). Among the demographic covariates, lower income is associated with greater willingness to test in all models (*P*<.01 across models). Older individuals have increased testing intention in the multiple linear model (*P*=.02). Among the psychosocial factors, favorable “LINK attitudes” (*P*<.01 across models), “LINK engagement” (*P*<.05 across models), and “Identification with LINK brand” (*P*<.05 across model) were all associated with increased desire to obtain routine HIV testing through LINK.

Within the target area and secondary catchment, participants living in zip codes with fewer available HIV services (*P*<.001), lower HIV prevalence (*P*=.01), greater proportion of black/African Americans (*P*<.001), smaller proportion of those 24 years or older living in the community (*P*<.001), and reduced median household income (*P*=.02) were associated with increased desire to obtain HIV testing through LINK, after adjustment for individual factors (Table 5).

**Table 4:** Individual-level predictor models for HIV testing.

				Multiple linear model			Random intercept model		
Predictor				Coefficient (95% CI)	Standardized coefficient (95% CI)	*P* value	Coefficient (95% CI)	Standardized coefficient (95% CI)	*P* value
Intercept				8.41 (5.86 to 10.97)	8.92 (8.70 to 9.13)	<.001	8.12 (5.65 to 10.75)	8.72 (8.05 to 9.38)	<.001
**Individual**									
							
		Race (white ref)		0.07 (-1.13 to 1.26)	0.01 (-0.20 to 0.22)	.91	0.14 (-1.02 to 1.31)	0.02 (-0.18 to 0.23)	.81
		Income^a^		-0.59 (-0.97 to -0.21)	-0.40 (-0.67 to 0.14)	.003	-0.60 (-0.97 to -0.23)	-0.41 (-0.66 to 0.16)	.002
		**Gender (Male ref)**							
			Female	0.20 (-0.25 to 0.64)	0.10 (-0.12 to 0.32)	.38	0.22 (-0.21 to 0.66)	0.11 (-0.11 to 0.33)	.32
			Transgender	0.83 (-0.29 to 1.94)	0.17 (-0.06 to 0.39)	.15	0.71 (-0.38 to 1.80)	0.14 (-0.08 to 0.36)	.20
		Age (years)		0.02 (0.01 to 0.40)	0.23 (0.01 to 0.44)	.04	0.02 (-0.02 to 0.37)	0.19 (-0.02 to 0.41)	.08
**Psychosocial**									
	LINK attitudes			0.38 (0.16 to 0.60)	0.36 (0.15 to 0.58)	<.001	0.35 (0.13 to 0.57)	0.34 (0.13 to 0.55)	.002
	LINK engagement			0.31 (0.09 to 0.53)	0.31 (0.09 to 0.53)	.006	0.30 (0.09 to 0.52)	0.30 (0.08 to 0.52)	.006
	Negative participatory norms			-0.00 (-0.23 to 0.22)	-0.00 (-0.23 to 0.22)	.98	-0.03 (-0.25 to 0.19)	-0.03 (-0.25 to 0.19)	.78
	LINK social support			0.09 (-0.14 to 0.32)	0.09 (-0.14 to 0.32)	.44	0.09 (-0.14 to 0.31)	0.08 (-0.14 to 0.31)	.45
	LINK Brand Perc.			-0.02 (-0.24 to 0.21)	-0.02 (-0.23 to 0.20)	.89	0.00 (-0.21 to 0.22)	0.00 (-0.21 to 0.22)	.97

^a^Income recorded in US $20K categories from 1 (<US $20,000) to 6 (>US $100,000).

**Table 5:** Model parameters for individual-level predictor models for HIV testing.

Model	N	Listwise deleted	σ_u_ (95% CI)	σ_e_ (95% CI)	Rho (95% CI)	LR test of σ_u_=0 (*P*)	AIC	BIC
Multiple linear model	421	63	—	—	—	—	1543	1586
Random intercept model	421	63	0.54 (0.09 to 3.14)	2.00 (1.86 to 2.16)	0.07 (0.00 to 0.59)	.05	1545	1595

**Table 6:** Multilevel predictor models for HIV Testing.^a^

	Neighborhood variable			Model parameters		
Adjusted Neighborhood-Level Predictor^b^	Coefficient (95% CI)	Standard. Coefficient (95% CI)	*P*	Rho (95%CI)	LR test of σ_u_=0 (*P*)	AIC
Number of HIV support services	-0.05 (-0.08 to -0.03)	-0.39 (-0.59 to -0.19)	<.001	0	>0.99	1535
HIV prevalence	-0.61 (-0.11 to -0.01)	-0.32 (-0.58 to -0.06)	.01	0.03 (0.00 to 0.16)	.008	1541
Black/African American population (%)	0.02 (0.01 to 0.04)	0.36 (0.14 to 0.57)	<.001	0	1.00	1539
Age ≥ 25 years (%)	-0.08 (-0.13 to -0.03)	-0.39 (-0.62 to -0.15)	<.001	0	1.00	1539
Male high-school graduation rate (%)	-0.08 (-0.17 to 0.00)	-0.26 (-0.53 to 0.00)	.05	0.02 (0.00 to 0.16)	.07	1543
Male employment (%)	-0.03 (-0.06 to 0.01)	-0.25 (-0.55 to 0.06)	.11	0.01	.14	1543
Median household income (US $K)	-0.07 (-0.13 to -0.01)	-0.34 (-0.61 to 0.06)	.02	0.01	.16	1540
Vacant homes (%)	0.04 (-0.06 to 0.14)	0.16 (-0.21 to 03.53)	.41	0.01	.12	1545

^a^Each multilevel model includes a single neighborhood-level variable together with all individual and psychosocial variables (N=421; 63 listwise deleted).

^b^Adjusted for race, income, gender, age, and the 5 psychosocial variables

### Participant Network Referral

Along with HIV testing intentions, participants also expressed a strong desire to refer other persons to LINK (mean 8.81 on 10-point scale [SD 1.86]). One-way ANOVA found significant differences in willingness to refer others among participants living in different areas (*F*_2,397_=5.812, *P*=.003). Tamhane post hoc analysis indicated that participants residing in the 2 CBI target zipcodes expressed greater intention to refer others to LINK than those residing outside the target and secondary catchment zip codes (difference=0.8, *P*=.01).

**Figure 3:**
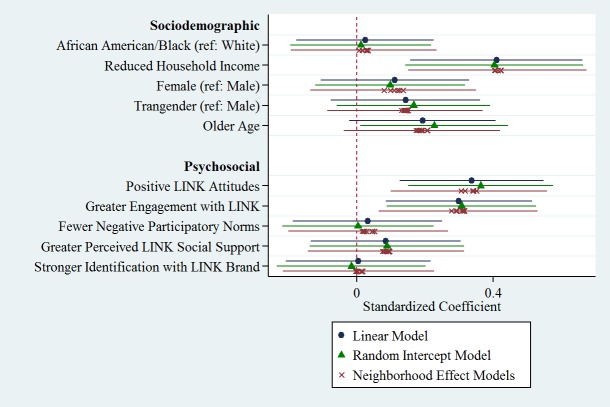
Individual predictors of CBI Referral.

**Figure 4:**
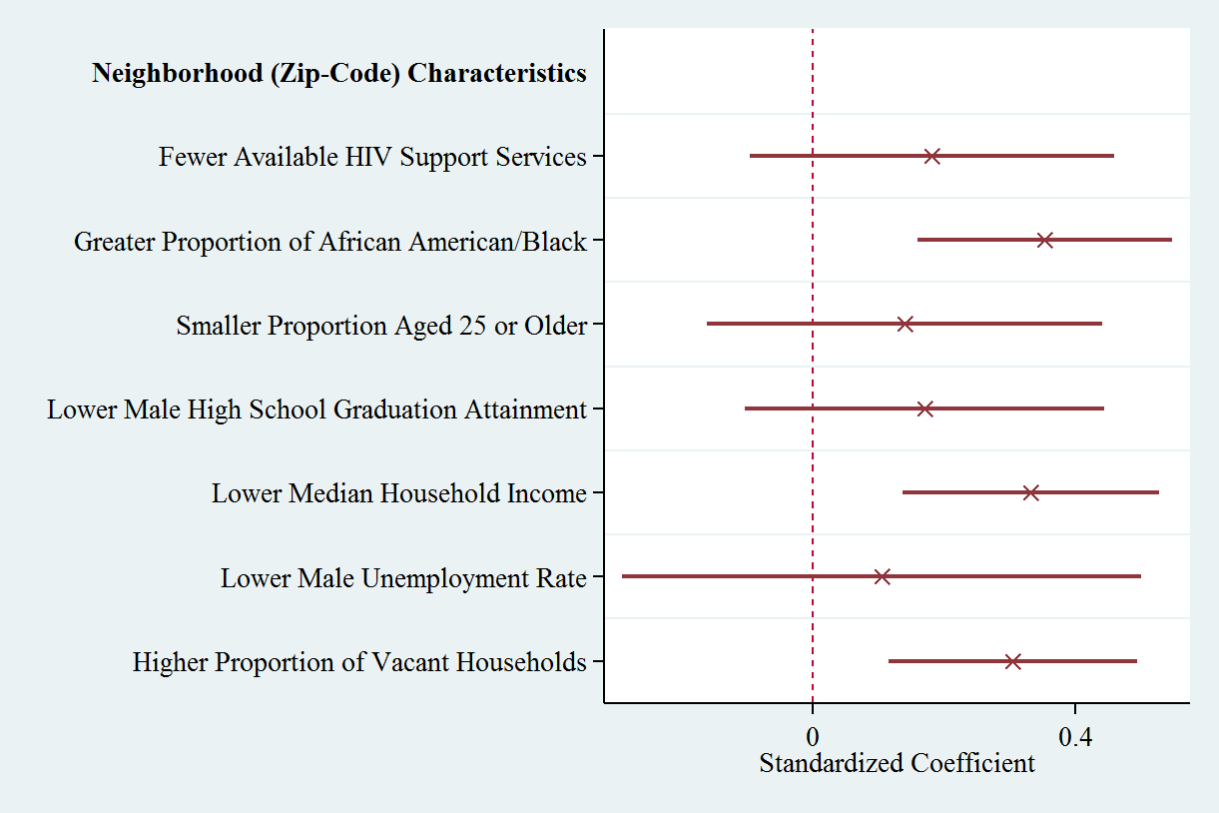
Adjusted neighborhoods predictors of CBI Referral.

The results of individual-level multivariable and random-intercepts models for CBI referral are detailed in Table 7 and model parameters in Table 8; multilevel neighborhood-factor models are provided in Table 9. Figures 3 and 4 show individual predictors of CBI referral and adjusted neighborhoods predictors of CBI referral, respectively. All models incorporate all individual and psychosocial covariates (race, income, gender, age, and the 5 psychosocial scales). The random-intercepts model further includes a random intercept for zip code, and the multilevel neighborhood-factor models include random intercepts as well as 1 neighborhood factor in each model. Full results for individual and psychosocial covariates in the multilevel models are given in Multimedia Appendix 1. The likelihood ratio test for null variance of the random intercept was statistically significant for referral to LINK services (*P*<.01 across models; Table 7). Individual-level coefficients are similar among these models (Tables 7 and 9). The results demonstrate that transgender individuals were more willing than men to refer members of their social network to LINK services (*P*<.05 across models). Participants living in zip codes with greater proportion of black/African Americans living in the CBI area (*P*<.001), greater proportion of vacant homes (*P*=.002), and reduced median household income (*P*<.001) were associated with increased desire to initiate referrals to LINK.

**Table 7:** Individual-level predictor models for HIV service referral.

			Multiple linear model			Random intercept model		
Predictor			Coefficient (95% CI)	Standardized Coefficient (95% CI)	*P*	Coefficient (95% CI)	Standardized Coefficient (95% CI)	*P*
Intercept			9.07 (6.79 to 11.35)	8.90 (8.71 to 9.10)	<.001	8.94 (6.72 to 11.17)	8.89 (8.49 to 9.30)	<.001
**Individual**								
	Race (white ref)		-0.32 (-0.41 to 0.76)	-0.06 (-0.24 to 0.13)	.56	-0.21 (-1.25 to 0.84)	-0.04 (-0.22 to 0.14)	.70
	Income^a^		-0.10 (-0.36 to 2.39)	-0.07 (-0.25 to 0.12)	.48	-0.10 (-0.35 to 1.67)	-0.06 (-0.24 to 0.11)	.49
	**Gender (male ref)**							
		Female	0.28 (-0.12 to 0.67)	0.14 (-0.06 to 0.34)	.17	0.35 (-0.03 to 0.74)	0.18 (-0.02 to 0.37)	.07
		Transgender	1.35 (0.31 to 2.39)	0.27 (0.06 to 0.48)	.01	1.28 (0.28 to 2.28)	0.26 (0.06 to 0.46)	.01
	Age (years)		0.08 (-0.10 to 0.26)	0.09 (-0.11 to 0.29)	.40	0.05 (-0.13 to 0.22)	0.05 (-0.14 to 0.24)	.61
**Psychosocial**								
	LINK attitudes		0.32 (0.11 to 0.53)	0.31 (0.11 to 0.51)	.003	0.30 (0.10 to 0.51)	0.29 (0.10 to 0.49)	.004
	LINK engagement		0.36 (0.16 to 0.56)	0.36 (0.16 to 0.55)	<.001	0.36 (0.17 to 0.55)	0.36 (0.17 to 0.54)	<.001
	Negative participatory norms		-0.12 (-0.31 to 0.79)	-0.12 (-0.32 to 0.08)	.24	-0.13 (-0.32 to 0.06)	-0.13 (-0.32 to 0.06)	.19
	LINK social support		0.20 (-0.15 to 0.41)	0.19 (-0.02 to 0.40)	.07	0.19 (-0.02 to 0.39)	0.18 (-0.02 to 0.38)	.07
	LINK brand Perception		0.22 (0.03 to 0.40)	0.21 (0.03 to 0.39)	.02	0.22 (0.04 to 0.40)	0.22 (0.04 to 0.39)	.02

^a^Income recorded in US $20K categories from 1 (<US $20,000) to 6 (>US $100,000).

**Table 8:** Model parameters for individual-level predictor models for HIV service referral.

Model	N	Listwise deleted	σ_u_ (95% CI)	σ_e_ (95% CI)	Rho (95% CI)	LR test of σ_u_=0 (*P*)	AIC	BIC
Multiple linear model	451	145	—	—	—	—	1198	1239
Random intercept model	451	145	0.31 (0.10 to 0.97)	1.62 (1.50 to 1.76)	0.03 (0.00 to 0.21)	.008	1196	1244

**Table 9:** Multilevel predictor models for HIV service referral.^a^

	Neighborhood variable			Model parameters		
Adjusted Neighborhood-Level Predictor^b^	Coefficient (95% CI)	Standardized Coefficient (95% CI)	*P*	Rho (95% CI)	LR test of σ_u_=0 (*P*)	AIC
Number of HIV support services	-0.02 (-0.06 to 0.01)	-0.18 (-0.46 to 0.10)	.20	0.01 (0.00 to 0.27)	.27	1196
HIV prevalence	-0.02 (-0.06 to 0.03)	-0.08 (-0.33 to 0.17)	.53	0.04	.009	1197
Black/African American population (%)	0.02 (0.01 to 0.04	0.35 (0.16 to 0.55)	<.001	0.00	>.99	1191
Age ≥ 25 years (%)	-0.03 (-0.09 to 0.03)	-0.14 (-0.44 to 0.16)	.36	0.02 (0.00 to 0.21)	.11	1197
Male high-school graduation	-0.05 (-0.14 to 0.03)	-0.17 (-0.44 to 0.10)	.22	0.03 (0.00 to 0.16)	.006	1196
Male employment (%)	-0.01 (-0.05 to 0.03)	-0.11 (-0.50 to 0.29)	.60	0.02 (0.00 to 0.27)	.17	1197
Median household income (US $K)	-0.07 (-0.11 to -0.03)	-0.33 (-0.53 to 0.14)	<.001	0.00	>.99	1193
Vacant homes (%)	0.08 (0.03 to 0.13)	0.31 (0.12 to 0.49)	.002	0.00	>.99	1194

^a^Each multi-level model includes a single neighborhood-level variable together with all individual and psychosocial variables (N=451; 145 listwise deleted).

^b^Adjusted for race, income, gender, age, and the 5 psychosocial variables.

## Discussion

### Summary of Main Findings

This study found that Project LINK participants were extremely positive about their experience with the CBI, and expressed high intentions to use LINK resources to obtain an HIV test and to refer others to the CBI. Both of these outcomes were significantly higher for participants living in target areas compared with those living outside the target and adjoining regions. In addition, for participants in the target and secondary catchment areas, several individual- and neighborhood-level factors were found to be associated with intentions to use LINK resources for HIV testing and with desire to refer others to the CBI. Reduced income, older age, positive attitudes about the CBI, and increased engagement with LINK were all associated with increased desire to use LINK testing resources. On the neighborhood level, intention to test with LINK was associated with reduced availability of support services, reduced HIV prevalence, greater proportion of black/African Americans in the neighborhood, reduced proportion of adults aged 24 years or older, and reduced median household income. Desire to refer others to Project LINK was found to be associated with transgender individuals, positive attitudes about LINK, LINK engagement, identification with the LINK brand, greater proportion of black/African Americans in the neighborhood, reduced median neighborhood household income, and a greater proportion of vacant homes in the neighborhood.

### Interpretations and Comparison With Prior Work

These results suggests that Project LINK successfully contributed to HIV testing intentions and HIV service referral among those living in a geographically targeted area characterized by high poverty and HIV prevalence and low availability of HIV services [12]. Greater intention to test and engage others in testing and care referral are critically important outcomes for the realization of continuum of care objectives such as normalized population-level HIV testing, and subsequent referral to and prompt entry into care [9,34].

Living in high-stress neighborhood conditions has been associated with increased risk for HIV/sexually transmitted infection transmission [35], sexual risk behavior, and substance abuse [36]. Recognizing that these factors have contributed to the high HIV prevalence in the LINK-targeted neighborhoods, the CBI partners sought to develop an intervention that was relatively easy to implement through mobile delivery of their existing services to improve community access to diagnosis and care. As a result, those living in the intervention area could select from a menu of 24/7 LINK offerings such as partner-delivered medical and mental health services, domestic violence support groups, family counseling, and individual or group-based “Healthy Love” sexual health and well-being training, all delivered in familiar settings such as homes, schools, churches, community centers, and corner “store front” organizations.

The findings suggest that Project LINK events successfully attracted the enthusiastic participation of the community it served, including those challenged by the environment they lived in that was characterized by poverty, transportation limitations, and high unemployment. Among the psychosocial factors assessed, we found that positive attitudes about HIV testing and the CBI are critically important for facilitating linkage to testing and referrals to the CBI services.

Notably, LINK drew the participation of black/African American transgender women (transwomen) who represent a highly vulnerable group for HIV transmission [37]. With an estimated 60% of annual HIV infections diagnosed among MSM and transwomen, LINK’s culturally sensitive service delivery approach appeared to resonate with the transgender population [38]. The data indicate that transgender individuals showed significantly higher intention to refer others to LINK, even after adjustment for other individual-, psychosocial-, and neighborhood-level covariates. The sample of transgender individuals was small (n=27), so we are cautious of generalizing this result, but we feel that it indicates this CBI model's success in providing a safe and comfortable service environment for those who are marginalized and/or disenfranchised with existing health care options.

Distal neighborhood-level factors played a role in promoting HIV testing intentions and referral to the CBI services [39]. Stronger outcomes were observed among those living in predominately black/African American tracts. We believe these factors can be linked to the messages delivered by LINK partners and others that black/African Americans are disproportionately affected by HIV/AIDS and account for a higher proportion of people living with HIV at every stage from new infection to death [40]. Thus, living in an area with a high case rate among black/African Americans alters individuals’ perceptions of their perceived vulnerability to HIV and motivates testing and referral to care and treatment services [41].

We also observed a strong indicator that neighborhood disorder, as evidenced by the density of nearby vacant households, is associated with HIV testing and referral [42]. We assert that this may be indicative of the presence of drug activity including injection drug use and extent of “crack houses” in the neighborhood, both associated with greater concentration of HIV risk behavior and prevalent cases [43-45]. Our study corresponds with previous evidence that intravenous drug users living in lower income areas may be more likely to utilize neighborhood prevention programs than those living in more affluent areas [46]. Thus, our findings evidence the need for targeted community interventions such as LINK in similar high-stress neighborhoods, and reflect the efficacy of LINK in reaching these neighborhoods.

On the individual level, those persons facing the extreme poverty were more likely to intend to obtain HIV testing. There was no difference in testing intentions between men and women, or between white and black/African American participants. Because intention to utilize LINK resources for testing was high on average, especially in the primary target area, this reflects the successful targeting of individuals with the highest need for linkage to care, regardless of race or gender. Although the small number of zip codes in our sample does not allow for effective separation of the neighborhood-level effect across heterogeneous neighborhoods, the large number of significant neighborhood-level factors suggests that community structure plays an important role in the success of targeted HIV care linkage interventions. Among those participants residing in the target area, the reduced availability of local HIV support services resulted in much greater willingness to use LINK for HIV testing. In this respect, we argue that LINK offers a model for reaching historically marginalized populations through its geographically focused, socially compatible service delivery approach [2]. The results also reflect the reality of effectively promoting HIV prevention in communities with considerable challenges; our theoretical orientation was validated by the findings that suggested direct and indirect effects of multiple levels of influence on HIV testing intentions and referral patterns.

The results of this project indicate the importance of including CBIs as options for HIV prevention planning that seeks to increase access to HIV testing and delay the time for linkage to care. Timely linkage to and retention in care is key to ensuring that patients living with HIV reach an undetectable status, which in turns helps to decrease the transmission of new infections. Furthermore, decreasing the waiting time for accessing care also increases the likelihood of people actually beginning therapy, and increasing their chances for improved health while living with HIV. Finally, the outcomes of the project support ensuring that communities and their members are not only engaged when it comes to accessing HIV testing, but are also engaged in the planning, site selection, and coordination of both HIV testing events and activities, as well as strategic planning to ensure linkage to care for any services that will take place within their communities [47]. Thus, CBIs should continue to be used for prevention work in communities, especially when targeting improvements in HIV testing and linkage to care. Linkage strategies should consider incorporating community-level engagement to support timely linkage and retention in care. Bringing services into communities and enabling easy access to neighborhood-based services closer may help reach the immediate linkage-to-care goals of the National AID Strategy and the CDC’s High-Impact Prevention programmatic policy.

### Limitations

We recognize the limitations associated with self-report, which is susceptible to social desirability bias. In addition, our random sampling approach within venues hosting LINK activities and events is effective for describing associations within the population of attendees [48]. However, this sampling technique together with the locality of the study restricts the ability to generalize results to other neighborhoods, cities, or to nonattendees. Furthermore, privacy concerns limited available address information to only zip-code-level data. While census tracts are generally considered the gold standard for neighborhood-level analysis, zip codes have been successfully used to characterize neighborhood factors in many recent studies in which census-tract-level data are unavailable [49-52]. Although this pilot initiative demonstrated feasibility and acceptability of the concept utilizing attitudinal and intention data, no behavioral data on actual testing were collected. This is an important next step in examining the efficacy of this project.

### Conclusions

Project LINK used a targeted approach to reach marginalized populations that prompted greater intention to engage in HIV testing, care, and referral to community partner organizations serving those in the selected neighborhoods. Our study findings indicate that community/neighborhood and psychosocial factors are critical to future efforts to increase routine HIV testing intentions in underserved areas and enhance efforts for subsequent referral to care. Yet, this project also highlights the need for additional, long-term assessment to evidence the impact of CBIs on care continuum outcomes. The results from this study demonstrate the promise of CBIs to reach individuals within a 1st year of diagnosis for subsequent improvements in HIV-related health outcomes.

## Appendix

Multimedia Appendix 1 Results of multilevel models for HIV testing and CBI referral.

## Abbreviations

AHRC: Atlanta Harm Reduction Coalition
AIDS: acquired immune deficiency syndrome
ANOVA: analysis of variance
CBI: Community-based intervention
CBWW: The Center for Black Women's Wellness
CDC: Centers for Disease Control and Prevention
HIV: human immunodeficiency virus
MCAR: Missing Completely at Random
MSM: men who have sex with men

## References

[1] Salam RA, Haroon S, Ahmed HH, Das JK, Bhutta ZA (2014). Impact of community-based interventions on HIV knowledge, attitudes, and transmission. Infect Dis Poverty.

[2] Nunn A, Yolken A, Cutler B, Trooskin S, Wilson P, Little S, Mayer K (2014). Geography should not be destiny: Focusing HIV/AIDS implementation research and programs on microepidemics in US neighborhoods. Am J Public Health.

[3] Chen NE, Gallant JE, Page KR (2012). A systematic review of HIV/AIDS survival and delayed diagnosis among Hispanics in the United States. J Immigr Minor Health.

[4] MacKellar DA, Valleroy LA, Secura GM, Behel S, Bingham T, Celentano DD, Koblin BA, Lalota M, McFarland W, Shehan D, Thiede H, Torian LV, Janssen RS, Young Men's Survey Study Group (2005). Unrecognized HIV infection, risk behaviors, and perceptions of risk among young men who have sex with men: Opportunities for advancing HIV prevention in the third decade of HIV/AIDS. J Acquir Immune Defic Syndr.

[5] Torian LV, Wiewel EW, Liu K, Sackoff JE, Frieden TR (2008). Risk factors for delayed initiation of medical care after diagnosis of human immunodeficiency virus. Arch Intern Med.

[6] Tripathi A, Gardner LI, Ogbuanu I, Youmans E, Stephens T, Gibson JJ, Duffus WA (2011). Predictors of time to enter medical care after a new HIV diagnosis: A statewide population-based study. AIDS Care.

[7] Centers for Disease Control and Prevention (2015). HIV Surveillance Supplemental Report.

[8] Anthony MN, Gardner L, Marks G, Anderson-Mahoney P, Metsch LR, Valverde EE, Del RC, Loughlin AM, Antiretroviral TreatmentAccess Study (ARTAS) Study Group (2007). Factors associated with use of HIV primary care among persons recently diagnosed with HIV: Examination of variables from the behavioural model of health-care utilization. AIDS Care.

[9] Gardner EM, McLees MP, Steiner JF, Del RC, Burman WJ (2011). The spectrum of engagement in HIV care and its relevance to test-and-treat strategies for prevention of HIV infection. Clin Infect Dis.

[10] Denning P, DiNenno E (2013). Communities in Crisis: Is There a Generalized HIV Epidemic in Impoverished Urban Areas of the United States? Who's At Risk for HIV.

[11] Shepard CW, Gortakowski HW, Nasrallah H, Cutler BH, Begier EM (2011). Using GIS-based density maps of HIV surveillance data to identify previously unrecognized geographic foci of HIV burden in an urban epidemic. Public Health Rep.

[12] Hixson BA, Omer SB, del RC, Frew PM (2011). Spatial clustering of HIV prevalence in Atlanta, Georgia and population characteristics associated with case concentrations. J Urban Health.

[13] Fulcher C, Kaukinen C (2005). Mapping and visualizing the location HIV service providers: An exploratory spatial analysis of Toronto neighborhoods. AIDS Care.

[14] Oppong JR, Tiwari C, Ruckthongsook W, Huddleston J, Arbona S (2012). Mapping late testers for HIV in Texas. Health Place.

[15] Eberhart MG, Yehia BR, Hillier A, Voytek CD, Blank MB, Frank I, Metzger DS, Brady KA (2013). Behind the cascade: Analyzing spatial patterns along the HIV care continuum. J Acquir Immune Defic Syndr.

[16] Philbin MM, Tanner AE, DuVal A, Ellen JM, Xu J, Kapogiannis B, Bethel J, Fortenberry JD, Adolescent Trials Network for HIV/AIDS Interventions (2014). Factors affecting linkage to care and engagement in care for newly diagnosed HIV-positive adolescents within fifteen adolescent medicine clinics in the United States. AIDS Behav.

[17] Krieger N (2001). Theories for social epidemiology in the 21st century: An ecosocial perspective. Int J Epidemiol.

[18] Parker EA, Chung LK, Israel BA, Reyes A, Wilkins D (2010). Community organizing network for environmental health: Using a community health development approach to increase community capacity around reduction of environmental triggers. J Prim Prev.

[19] Schultz Annette SH, Temple B, Gibbons C, Preston J, Ronson G (2014). Listening to those who are living with HIV and tobacco dependence and exploring their health care context. J Assoc Nurses AIDS Care.

[20] Frew PM, Archibald M, Hixson B, del RC (2011). Socioecological influences on community involvement in HIV vaccine research. Vaccine.

[21] (2011). High-Impact HIV Prevention: CDC's Approach to Reducing HIV Infections in the United States.

[22] Painter TM, Herbst JH, Diallo DD, White LD, Centers for Disease Control and Prevention (CDC) (2014). Community-based program to prevent HIV/STD infection among heterosexual black women. MMWR Surveill Summ.

[23] Diallo DD, Moore TW, Ngalame PM, White LD, Herbst JH, Painter TM (2010). Efficacy of a single-session HIV prevention intervention for black women: A group randomized controlled trial. AIDS Behav.

[24] Maher L, Iversen J, Kaldor J (2006). Measuring effectiveness of needle and syringe exchange programs for prevention of HIV among injecting drug users: Response to Amundsen. Addiction.

[25] Amundsen EJ (2006). Measuring effectiveness of needle and syringe exchange programmes for prevention of HIV among injecting drug users. Addiction.

[26] Holstad MM, Essien JE, Ekong E, Higgins M, Teplinskiy I, Adewuyi MF (2012). Motivational groups support adherence to antiretroviral therapy and use of risk reduction behaviors in HIV positive Nigerian women: A pilot study. Afr J Reprod Health.

[27] Rothenberg KH, Paskey SJ, Reuland MM, Zimmerman SI, North RL (1995). Domestic violence and partner notification: Implications for treatment and counseling of women with HIV. J Am Med Womens Assoc.

[28] Frew PM, Hou SI, Davis M, Chan K, Horton T, Shuster J, Hixson B, del RC (2010). The likelihood of participation in clinical trials can be measured: The Clinical Research Involvement Scales. J Clin Epidemiol.

[29] Pawinski R (2001). Community attitudes to HIV/AIDS. S Afr Med J.

[30] Miner MH, Peterson JL, Welles SL, Jacoby SM, Rosser BR (2009). How do social norms impact HIV sexual risk behavior in HIV-positive men who have sex with men?: Multiple mediator effects. J Health Psychol.

[31] Kumar S, Liu M, Hwang S (2012). A multifaceted comparison of ArcGIS and MapMarker for automated geocoding. Geospat Health.

[32] (2000). Census 2000.

[33] Boston RC, Sumner AE (2003). STATA: A statistical analysis system for examining biomedical data. Adv Exp Med Biol.

[34] Gardner EM, Daniloff E, Thrun MW, Reirden DH, Davidson AJ, Johnson SC, Wilmoth R, Connick E, Burman WJ (2013). Initial linkage and subsequent retention in HIV care for a newly diagnosed HIV-infected cohort in Denver, Colorado. J Int Assoc Provid AIDS Care.

[35] Kerr JC, Valois RF, Siddiqi A, Vanable P, Carey MP, DiClemente RJ, Romer D, Brown LK, Farber NB, Salazar LF (2015). Neighborhood condition and geographic locale in assessing HIV/STI risk among African American adolescents. AIDS Behav.

[36] Bowleg L, Neilands TB, Tabb LP, Burkholder GJ, Malebranche DJ, Tschann JM (2014). Neighborhood context and Black heterosexual men's sexual HIV risk behaviors. AIDS Behav.

[37] Garofalo R, Deleon J, Osmer E, Doll M, Harper GW (2006). Overlooked, misunderstood and at-risk: Exploring the lives and HIV risk of ethnic minority male-to-female transgender youth. J Adolesc Health.

[38] Centers for Disease Control and Prevention (2013). Centers for Disease Control and Prevention.

[39] Raymond HF, Chen Y, Syme SL, Catalano R, Hutson MA, McFarland W (2014). The role of individual and neighborhood factors: HIV acquisition risk among high-risk populations in San Francisco. AIDS Behav.

[40] Centers for Disease Control and Prevention (2013). HIV among African-Americans.

[41] Axelrad JE, Mimiaga MJ, Grasso C, Mayer KH (2013). Trends in the spectrum of engagement in HIV care and subsequent clinical outcomes among men who have sex with men (MSM) at a Boston community health center. AIDS Patient Care STDS.

[42] McCurdy SA, Ross MW, Kilonzo GP, Leshabari MT, Williams ML (2006). HIV/AIDS and injection drug use in the neighborhoods of Dar es Salaam, Tanzania. Drug Alcohol Depend.

[43] Latkin CA, Curry AD, Hua W, Davey MA (2007). Direct and indirect associations of neighborhood disorder with drug use and high-risk sexual partners. Am J Prev Med.

[44] Latkin CA, German D, Hua W, Curry AD (2009). Individual-level influences on perceptions of neighborhood disorder: A multilevel analysis. J Community Psychol.

[45] Latkin CA, Williams CT, Wang J, Curry AD (2005). Neighborhood social disorder as a determinant of drug injection behaviors: A structural equation modeling approach. Health Psychol.

[46] Buchanan D, Shaw S, Teng W, Hiser P, Singer M (2003). Neighborhood differences in patterns of syringe access, use, and discard among injection drug users: Implications for HIV outreach and prevention education. J Urban Health.

[47] Centers for Disease Control and Prevention (2011). High-Impact HIV Prevention: CDC's Approach to Reducing HIV Infections in the United States.

[48] Muhib FB, Lin LS, Stueve A, Miller RL, Ford WL, Johnson WD, Smith PJ, Community Intervention Trial for Youth Study Team (2001). A venue-based method for sampling hard-to-reach populations. Public Health Rep.

[49] Brumback BA, Zheng HW, Dailey AB (2013). Adjusting for confounding by neighborhood using generalized linear mixed models and complex survey data. Stat Med.

[50] Lim J, Ashing-Giwa KT (2011). Examining the effect of minority status and neighborhood characteristics on cervical cancer survival outcomes. Gynecol Oncol.

[51] Sathyanarayanan S, Brooks AJ, Hagen SE, Edington DW (2012). Multilevel analysis of the physical health perception of employees: Community and individual factors. Am J Health Promot.

[52] Walker DR, Inglese GW, Sloand JA, Just PM (2010). Dialysis facility and patient characteristics associated with utilization of home dialysis. Clin J Am Soc Nephrol.

