# Supplementary material for: An Integrated Service Delivery Model to Identify Persons Living with HIV and to Provide Linkage to HIV Treatment and Care in Prioritized Neighborhoods: A Geotargeted, Program Outcome Study
Source: JMIR Public Health Surveill. 2015 Oct 8;1(2):e16. doi: 10.2196/publichealth.4675 (PMC4869208; doi:10.2196/publichealth.4675)
Supplement: Multimedia Appendix 1 [file publichealth_v1i2e16_app1.docx]

## Appendix 1: Results of multilevel models for HIV testing and CBI referral

#### Part 1: Multilevel models for HIV Testing

**Table 1: Multi-level predictor model for effect of number of HIV support services available in the zip code on HIV Testing**

| Predictor | | | | Coeff. (SE) | Std. Coeff.^a^ (SE) | *P-value* |
| --- | --- | --- | --- | --- | --- | --- |
|  | | | |  |  |  |
| *Intercept* | | | | 8.91 (1.26) | 8.92 (0.11) | .000 |
|  | | | |  |  |  |
| **Neighborhood** | | | |  |  |  |
|  | | Number of HIV Support Services | | -0.05 (0.01) | 0.39 (0.10) | .000 |
|  | |  | |  |  |  |
| **Individual** | | | |  |  |  |
|  | Race (White ref) | | | 0.09 (0.59) | 0.01 (0.10) | .89 |
|  | Income^b^ | | | -0.62 (0.19) | -0.42 (0.13) | .001 |
|  | *Gender (Male ref)* | | |  |  |  |
|  |  | | Female | 0.20 (0.22) | 0.10 (0.11) | .36 |
|  |  | | Transgender | 0.65 (0.55) | 0.13 (0.11) | .24 |
|  | Age (years) | | | 0.16 (0.10) | 0.18 (0.11) | .10 |
|  | | | |  |  |  |
| **Psychosocial** | | | |  |  |  |
|  | LINK Attitudes | | | 0.32 (0.11) | 0.31 (0.11) | .004 |
|  | LINK Engagement | | | 0.30 (0.11) | 0.29 (0.11) | .007 |
|  | Neg. Part. Norms | | | -0.05 (0.11) | -0.05 (0.11) | .64 |
|  | LINK Social Supp. | | | 0.08 (0.11) | 0.08 (0.11) | .47 |
|  | LINK Brand Perc. | | | 0.02 (0.11) | 0.02 (0.11) | .88 |
|  | | | |  |  |  |
| **Model Parameters** | | | |  |  |  |
|  | N | | | 421 |  |  |
|  | *Listwise deleted* | | | *63* |  |  |
|  | σ_u_ | | | 0.00 (0.11) |  |  |
|  | σ_e_ | | | 1.99 (0.07) |  |  |
|  | Rho | | | 0.00 (-) |  |  |
|  | LR test of σ_u_=0 | | | P=1.00 |  |  |
|  | AIC | | | 1535 |  |  |
|  | BIC | | | 1590 |  |  |

^a^Standardized Coefficients (Standard Error)

^b^Income recorded in $20K categories from 1= <$20K to 6=>$100K

**Table 2: Multi-level predictor model for effect of zip code HIV prevalence on HIV Testing**

| Predictor | | | | Coeff. (SE) | Std. Coeff.^a^ (SE) | *P-value* |
| --- | --- | --- | --- | --- | --- | --- |
|  | | | |  |  |  |
| *Intercept* | | | | 9.85 (1.44) | 8.85 (0.23) | .000 |
|  | | | |  |  |  |
| **Neighborhood** | | | |  |  |  |
|  | | HIV Prevalence (per 10,000) | | -0.06 (0.02) | -0.32 (0.13) | .014 |
|  | |  | |  |  |  |
| **Individual** | | | |  |  |  |
|  | Race (White ref) | | | 0.04 (0.59) | 0.01 (0.10) | .95 |
|  | Income^b^ | | | -0.62 (0.19) | -0.42 (0.13) | .001 |
|  | *Gender (Male ref)* | | |  |  |  |
|  |  | | Female | 0.16 (0.22) | 0.08 (0.11) | .47 |
|  |  | | Transgender | 0.68 (0.55) | 0.14 (0.11) | .21 |
|  | Age (years) | | | 0.16 (0.10) | 0.18 (0.11) | .09 |
|  | | | |  |  |  |
| **Psychosocial** | | | |  |  |  |
|  | LINK Attitudes | | | 0.33 (0.11) | 0.32 (0.11) | .003 |
|  | LINK Engagement | | | 0.28 (0.11) | 0.28 (0.11) | .01 |
|  | Neg. Part. Norms | | | -0.05 (0.11) | -0.05 (0.11) | .64 |
|  | LINK Social Supp. | | | 0.08 (0.11) | 0.08 (0.11) | .50 |
|  | LINK Brand Perc. | | | 0.02 (0.11) | 0.02 (0.11) | .88 |
|  | | | |  |  |  |
| **Model Parameters** | | | |  |  |  |
|  | N | | | 421 |  |  |
|  | *Listwise deleted* | | | *63* |  |  |
|  | σ_u_ | | | 0.36 (0.19) |  |  |
|  | σ_e_ | | | 1.99 (0.07) |  |  |
|  | Rho | | | 0.03 (0.03) |  |  |
|  | LR test of σ_u_=0 | | | P=0.008 |  |  |
|  | AIC | | | 1541 |  |  |
|  | BIC | | | 1595 |  |  |

^a^Standardized Coefficients (Standard Error)

^b^Income recorded in $20K categories from 1= <$20K to 6=>$100K

#### Part 2: Multilevel models for HIV Service Referral

**Table 3: Multilevel predictor model for effect of number of HIV support services available in the zip code on HIV Service Referral**

| Predictor | | | | Coeff. (SE) | Std. Coeff.^a^ (SE) | *P-value* |
| --- | --- | --- | --- | --- | --- | --- |
|  | | | |  |  |  |
| *Intercept* | | | | 9.32 (1.16) | 8.92 (0.15) | .000 |
|  | | | |  |  |  |
| **Neighborhood** | | | |  |  |  |
|  | | Number of HIV Support Services | | -0.02 (0.02) | 0.18 (0.14) | .20 |
|  | |  | |  |  |  |
| **Individual** | | | |  |  |  |
|  | Race (White ref) | | | -0.30 (0.54) | -0.05 (0.09) | .59 |
|  | Income^b^ | | | -0.09 (0.13) | -0.06 (0.09) | .47 |
|  | *Gender (Male ref)* | | |  |  |  |
|  |  | | Female | 0.32 (0.20) | 0.16 (0.10) | .12 |
|  |  | | Transgender | 1.25 (0.51) | 0.25 (0.10) | .02 |
|  | Age (years) | | | 0.04 (0.09) | 0.05 (0.10) | .65 |
|  | | | |  |  |  |
| **Psychosocial** | | | |  |  |  |
|  | LINK Attitudes | | | 0.36 (0.10) | 0.28 (0.10) | .006 |
|  | LINK Engagement | | | 0.14 (0.10) | 0.35 (0.10) | .000 |
|  | Neg. Part. Norms | | | -0.14 (0.10) | -0.14 (0.10) | .15 |
|  | LINK Social Supp. | | | 0.19 (0.10) | 0.19 (0.10) | .07 |
|  | LINK Brand Perc. | | | 0.23 (0.10) | 0.22 (0.09) | .01 |
|  | | | |  |  |  |
| **Model Parameters** | | | |  |  |  |
|  | N | | | 451 |  |  |
|  | *Listwise deleted* | | | *145* |  |  |
|  | σ_u_ | | | 0.17 (0.20) |  |  |
|  | σ_e_ | | | 1.63 (0.07) |  |  |
|  | Rho | | | 0.01 (0.03) |  |  |
|  | LR test of σ_u_=0 | | | P=.27 |  |  |
|  | AIC | | | 1196 |  |  |
|  | BIC | | | 1248 |  |  |

^a^Standardized Coefficients (Standard Error)

^b^Income recorded in $20K categories from 1= <$20K to 6=>$100K

**Table 4: Multi-level predictor model for effect of zip code HIV prevalence on HIV Service Referral**

| Predictor | | | | Coeff. (SE) | Std. Coeff.^a^ (SE) | *P-value* |
| --- | --- | --- | --- | --- | --- | --- |
|  | | | |  |  |  |
| *Intercept* | | | | 9.36 (1.32) | 8.91 (0.22) | .000 |
|  | | | |  |  |  |
| **Neighborhood** | | | |  |  |  |
|  | | HIV Prevalence (per 10,000) | | -0.02 (0.02) | 0.08 (0.13) | .53 |
|  | |  | |  |  |  |
| **Individual** | | | |  |  |  |
|  | Race (White ref) | | | -0.24 (0.54) | -0.04 (0.09) | .65 |
|  | Income^b^ | | | -0.10 (0.13) | -0.07 (0.09) | .46 |
|  | *Gender (Male ref)* | | |  |  |  |
|  |  | | Female | 0.34 (0.20) | 0.17 (0.10) | .08 |
|  |  | | Transgender | 1.27 (0.51) | 0.26 (0.10) | .01 |
|  | Age (years) | | | 0.04 (0.09) | 0.05 (0.10) | .64 |
|  | | | |  |  |  |
| **Psychosocial** | | | |  |  |  |
|  | LINK Attitudes | | | 0.30 (0.10) | 0.29 (0.10) | .005 |
|  | LINK Engagement | | | 0.36 (0.10) | 0.35 (0.10) | .000 |
|  | Neg. Part. Norms | | | -0.13 (0.10) | -0.13 (0.10) | .17 |
|  | LINK Social Supp. | | | 0.19 (0.10) | 0.19 (0.10) | .07 |
|  | LINK Brand Perc. | | | 0.22 (0.09) | 0.22 (0.09) | .01 |
|  | | | |  |  |  |
| **Model Parameters** | | | |  |  |  |
|  | N | | | 451 |  |  |
|  | *Listwise deleted* | | | *145* |  |  |
|  | σ_u_ | | | 0.33 (0.18) |  |  |
|  | σ_e_ | | | 1.62 (0.07) |  |  |
|  | Rho | | | 0.04 (0.04) |  |  |
|  | LR test of σ_u_=0 | | | P=0.009 |  |  |
|  | AIC | | | 1197 |  |  |
|  | BIC | | | 1250 |  |  |

^a^Standardized Coefficients (Standard Error)

^b^Income recorded in $20K categories from 1= <$20K to 6=>$100K

**Table 5: Multi-level predictor model for effect of percentage of zip code that is Black/African American on HIV Service Referral**

| Predictor | | | | Coeff. (SE) | Std. Coeff.^a^ (SE) | *P-value* |
| --- | --- | --- | --- | --- | --- | --- |
|  | | | |  |  |  |
| *Intercept* | | | | 7.16 (1.24) | 8.89 (0.09) | .000 |
|  | | | |  |  |  |
| **Neighborhood** | | | |  |  |  |
|  | | Black/African American Pop (%) | | 0.02 (0.01) | 0.35 (0.10) | .000 |
|  | |  | |  |  |  |
| **Individual** | | | |  |  |  |
|  | Race (White ref) | | | -0.25 (0.53) | -0.04 (0.09) | .64 |
|  | Income^b^ | | | -0.10 (0.13) | -0.07 (0.09) | .45 |
|  | *Gender (Male ref)* | | |  |  |  |
|  |  | | Female | 0.34 (0.19) | 0.17 (0.10) | .08 |
|  |  | | Transgender | 1.25 (0.51) | 0.25 (0.10) | .01 |
|  | Age (years) | | | 0.04 (0.09) | 0.04 (0.10) | .68 |
|  | | | |  |  |  |
| **Psychosocial** | | | |  |  |  |
|  | LINK Attitudes | | | 0.29 (0.10) | 0.28 (0.10) | .006 |
|  | LINK Engagement | | | 0.35 (0.10) | 0.35 (0.10) | .000 |
|  | Neg. Part. Norms | | | -0.14 (0.10) | -0.14 (0.10) | .14 |
|  | LINK Social Supp. | | | 0.18 (0.10) | 0.18 (0.10) | .08 |
|  | LINK Brand Perc. | | | 0.23 (0.09) | 0.22 (0.09) | .01 |
|  | | | |  |  |  |
| **Model Parameters** | | | |  |  |  |
|  | N | | | 451 |  |  |
|  | *Listwise deleted* | | | *145* |  |  |
|  | σ_u_ | | | 0.00 (0.10) |  |  |
|  | σ_e_ | | | 1.62 (0.07) |  |  |
|  | Rho | | | 0.00 (-) |  |  |
|  | LR test of σ_u_=0 | | | P=1.00 |  |  |
|  | AIC | | | 1191 |  |  |
|  | BIC | | | 1243 |  |  |

^a^Standardized Coefficients (Standard Error)

^b^Income recorded in $20K categories from 1= <$20K to 6=>$100K

**Table 6: Multi-level predictor model for effect of percentage of zip code age 25 or older on HIV Service Referral**

| Predictor | | | | Coeff. (SE) | Std. Coeff.^a^ (SE) | *P-value* |
| --- | --- | --- | --- | --- | --- | --- |
|  | | | |  |  |  |
| *Intercept* | | | | 10.69 (2.22) | 8.92 (0.18) | .000 |
|  | | | |  |  |  |
| **Neighborhood** | | | |  |  |  |
|  | | Age ≥25 years (%) | | -0.03 (0.03) | 0.14 (0.15) | .36 |
|  | |  | |  |  |  |
| **Individual** | | | |  |  |  |
|  | Race (White ref) | | | -0.25 (0.54) | -0.04 (0.09) | .64 |
|  | Income^b^ | | | -0.09 (0.13) | -0.06 (0.09) | .49 |
|  | *Gender (Male ref)* | | |  |  |  |
|  |  | | Female | 0.34 (0.20) | 0.17 (0.10) | .09 |
|  |  | | Transgender | 1.26 (0.51) | 0.25 (0.10) | .01 |
|  | Age (years) | | | 0.04 (0.09) | 0.05 (0.10) | .65 |
|  | | | |  |  |  |
| **Psychosocial** | | | |  |  |  |
|  | LINK Attitudes | | | 0.30 (0.10) | 0.29 (0.10) | .005 |
|  | LINK Engagement | | | 0.36 (0.10) | 0.36 (0.10) | .000 |
|  | Neg. Part. Norms | | | -0.13 (0.10) | -0.13 (0.10) | .17 |
|  | LINK Social Supp. | | | 0.19 (0.10) | 0.19 (0.10) | .07 |
|  | LINK Brand Perc. | | | 0.23 (0.09) | 0.22 (0.09) | .01 |
|  | | | |  |  |  |
| **Model Parameters** | | | |  |  |  |
|  | N | | | 451 |  |  |
|  | *Listwise deleted* | | | *145* |  |  |
|  | σ_u_ | | | 0.24 (0.18) |  |  |
|  | σ_e_ | | | 1.63 (0.07) |  |  |
|  | Rho | | | 0.02 (0.03) |  |  |
|  | LR test of σ_u_=0 | | | P=0.11 |  |  |
|  | AIC | | | 1197 |  |  |
|  | BIC | | | 1249 |  |  |

^a^Standardized Coefficients (Standard Error)

^b^Income recorded in $20K categories from 1= <$20K to 6=>$100K
